# 3D Printing of Meat Following Supercritical Fluid Extraction

**DOI:** 10.3390/foods11040554

**Published:** 2022-02-15

**Authors:** Abhilash Aditya, Namsoo Peter Kim

**Affiliations:** 1 Center for Printable Materials Certificate, The University of Texas at El Paso, EL Paso, TX 79968, USA; aaditya@utep.edu; 2 Department of Metallurgical Materials and Biomedical Engineering, The University of Texas at El Paso, EL Paso, TX 79968, USA; 3 Brain Pool Program, Korea University, Seoul 02841, Korea

**Keywords:** supercritical fluid (SCF), 3D-printed meat, untact food delivery

## Abstract

With the spread of COVID-19, understanding the spread of food poisoning, managing food materials related to chronic diseases, food ingredients’ reliability, and non-face-to-face or untact delivery methods are rapidly emerging. A new field of meat research has been introduced for hygienic and healthy recipes to maintain freshness and control personalized ingredients using supercritical processes and 3D printing technology. Supercritical fluid extraction processes (SCF) and untact 3D printing technology will replace traditional meat freshness assessment based on color change according to the degree of oxidation of myoglobin in meat. SCF processes safely and quickly remove residual blood from meat and control fat and cholesterol that may be harmful to the human body. SCF-processed, high-viscosity meats are printed remotely through repeated IoT system variable experiments in WEB-CLOUD between UTEP in Texas, USA, and Korea University in Seoul, Korea. The SCF process in this study confirmed a weight reduction of 8.5% to 22.5%, depending on the temperature, pressure, and SCF process time. Under conditions of a tip size of 1.0 × 10^−3^ m, a shear rate of 200/s, and a maximum pressing force of 170 N, a 1000 cm^3^ SCF-processed meat was successfully 3D printed at the other site by transmitting G-code through web.

## 1. Introduction

There are many problems with the traditional assessment of the freshness of meat using the degree of oxidation by myoglobin. Myoglobin increases with muscle mass and age, and for beef, the inside turns bright red when the internal temperature is 60 °C, and when red meat is cooked at 60 to 70 °C, the pink darkens and becomes grayish brown. The oxidation of proteins, including myoglobin, exhibits various colors depending on heat and the degree of oxidation [1]. This study implements a method of maintaining freshness using an SCF process capable of 3D printing by removing residual blood, fat, and softening the texture. The Food and Agriculture Organization of the United States (FAO) recommends cooking meat for at least 3 min at an internal temperature of at least 63 °C to kill bacteria [2]. As the temperature rises, a red liquid containing myoglobin and water is extracted from the meat, making it bad for hygiene and aesthetics. There is a movement to create healthy dishes across societies where people are increasingly interested in maintaining a diet and monitoring nutrition by eating organic and hygienic foods [3,4,5]. Lately, the realization of personalized diet benefits via the accurate and fast printing of hygienic food demonstrates the potential of the technology in food engineering [6,7]. This study proposes a new paradigm shift in food processing, aiding significant advancements in the food industry [8,9,10]. This study tries to provide an SCF process and printable texture to remove bacteria at low temperatures. Apart from its religious significance, halal food is widely recognized as better quality food, attracting consumers for various reasons, from a healthy lifestyle perspective to restrictions on dairy products and other chemicals such as alcohol used in food preparation [11]. However, the unexpected surge in demand for halal foods and the lack of its regulations results in contamination during the delivery process [12,13,14,15,16]. Therefore, a technology that processes halal meat quickly, economically, and hygienically to remove certain ingredients is required. A technology that can receive significant attention from consumers with an excellent potential for processing unique food ingredients is necessary. The proposed SCF process for the safe handling of meat serves as an essential tool for food processing and refining [17]. The SCF process widely applied in the culinary industry so far is as follows. Table 1 illustrates the effective treatment methods used for the extraction of ingredients from various food materials [18,19,20,21]. The study focuses on providing a platform for scientifically applying the SCF extraction process of meat, followed by 3D printing.

Nevertheless, there is a requirement for advanced nutritional management to provide significant benefits for individuals with specific food allergies and dietary habits [9,24]. The convergence of the SCF process and 3D printing opens a new way to create customized hygienic food ingredients capable of precise control over nutritional composition. The SCF process enables faster residual blood extraction when juxtaposed with the traditional halal meat process. The study attempts the simultaneous extraction of cholesterol and residual blood, making the process efficient and economical. Moreover, 3D printing technology is one of the most anticipated engineering applications in the food industry.

Furthermore, SCF-processed foods possess porridge-like consistency, which is crucial as a building material in 3D printing. More specifically, the largest particle size is less than 5 × 10^−5^ m in diameter, and 3D printing of customized foods is possible if the moisture content is appropriately adjusted [25,26,27,28]. Some progress towards perfecting the 3D printing of meat ha become apparent, and soon the importance and scope of such a technique will be demonstrated [29,30]. 3D printing of food, meat, or meat substitutes requires specific textures critical to printability, and the SCF process can provide these essential textures and printability [31,32]. It is worth noting that the benefits of personalized food ingredients post-SCF-process and Internet of Things (IoT)-integrated 3D printing technology with a piston-type extruder (PTE) for high viscous printing can help children or the elderly who have difficulty swallowing food, further contributing to long-distance printing [33,34,35]. The results of this study provide critical information about processing parameters such as head speed, the required pressure drop, and the relationship between linear velocity and shear rate that is essential for the 3D printing of SCF-extracted meat.

## 2. Materials and Methods

### 2.1. Meat Preparation

Commercially available beef and chicken procured for the study underwent pre-experimental analysis, which resulted in no significant change in color and mass. However, the chicken was not subjected to the extraction process before 3D printing. A total of 5.00 ± 0.50 g of chicken and beef was pulverized at 6000~24,000 RPM over five minute cycles using a NINJA^®^ meat grinder. Three pulverization cycles were necessary and essential to maintain a size no larger than 5.0 × 10^−5^ m, to facilitate SCF extraction and allow better passage of CO_2_ into the meat. The pulverized chicken and beef were refrigerated at −20 °C in plastic bags to prevent meat degradation before experimentation.

### 2.2. SCF Extraction

**Figure 1:**
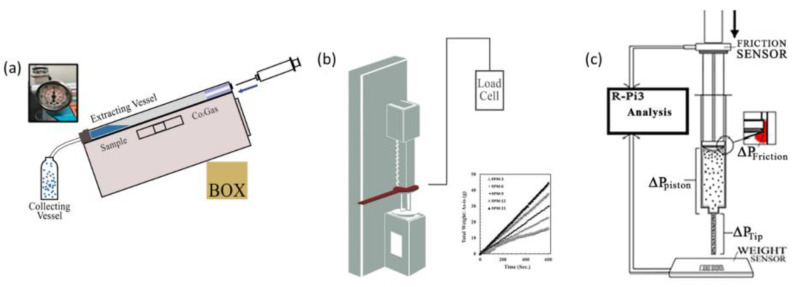
(**a**) A conceptual diagram of a supercritical extraction system, including a collection vessel, a sample extraction vessel, and a pressure monitor, and images of OCO-LABS parts. (**b**) IoT system control system including a weight control sensor. (**c**) Three-pressure drop concept diagram for 3D printing quantitative distribution.

SCF extraction utilizes an OCO-LAB apparatus shown in Figure 1a, where carbon dioxide (CO_2_) serves as the solvent. The extraction apparatus includes a collection vessel, a sample extraction vessel, and a pressure monitor. The processed meat and the extract are collected separately to quantify the extraction yield. The apparatus includes an extraction vessel with a maximum operating pressure of 5.07 × 10^7^ Pa. Pressure in the extraction vessel was kept constant by monitoring the gas pressure gauge in Figure 1c, and an internal sensor monitored the temperature. SCF extraction was performed at an inclination of about 45° to soak the sample in co-solvent and CO_2_ supercritical solvent. 10 mL of co-solvent was added by micropipette to the samples after being placed in an extraction vessel. The experimental conditions were as described in Table 2.

The extractions were conducted for 40 min to allow enough time for the SCF to dissolve the target components. Extractions were performed at 35 ± 5 °C or 50 ± 5 °C, using vegetable oil and water as co-solvents with CO_2_, respectively, and at three different pressures, 2.03 × 10^7^, 3.04 × 10^7^, and 4.05 × 10^7^ Pa. The experiment was conducted three times for each condition.

### 2.3. 3D Printing of Meat

The 3D printer utilized a piston-type extruder (PTE) with pressure sensors for precise control over the material flow, as shown in Figure 1b,c. Integration of IoT enabled remote and long-distance 3D printing. The experiments include 3D printing on-site at the University of Texas at El Paso, TX, USA, and Korea University, Seoul, Korea, demonstrating the capability of remote and long-distance 3D printing. The pressure required for the constant discharge of material and the pressure drop throughout the print were quantified through repeated experiments. The Raspberry Pi system installed in the printer recorded the pressure applied on the piston, the distance of the piston movement, and the weight of the material extruded in real-time. The experiments were conducted with air and water to acquire preliminary data, in order to elucidate the optimal printing process parameters for 3D printing meat. The forces caused by the friction between the components and the material accounts for the pressure drop. The friction between the piston and the inner wall of the cylinder (ΔP_Friction_), the resistance of the piston on the material (ΔP_Piston_), and the resistance offered by the nozzle tip to the extruded material (ΔP_Tip_) constitutes the pressure drop (ΔP_Total_) in real-time.

As SCF extraction efficiency was increased, the extracted meat inevitably resulted in a porridge-like texture. Conversely, the meat’s porridge-like texture is the most crucial factor in the quantitative control of 3D-printed foods. The clustered meat particles, solvent, and weight ratio of each were controlled to create the final printable fluid state. PTE tips ranging from 10^−4^ to 10^−2^ m allowed precise control and quantitative release for 3D meat printing. The 3D printer’s ability to print the material wth such consistency makes it capable of printing food with similar consistencies to, and not limited to, mashed vegetables, fish (sushi), and varieties of dough. The printing parameters used for halal chicken, halal beef, and SCF-processed beef were PTE head speeds in the range of 1.0 × 10^−3^–1.8 × 10^−2^ m/s, three different tip sizes, and indoor deposition temperature.

## 3. Results and Discussion

**Figure 2:**
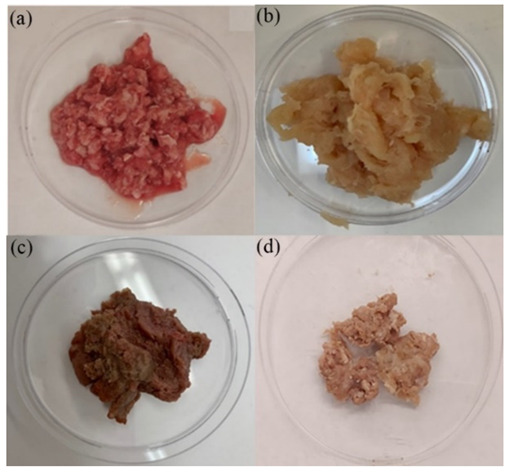
Samples of; (**a**): unprocessed beef; (**b**): unprocessed halal chicken; (**c**): unprocessed halal beef; (**d**): SC-CO_2_-processed beef under pressure at 3.04 × 10^7^ Pa and a temperature of 50 ± 5 °C.

The treatment process’s optimization needed three sets of experiments using a non-toxic, cost-effective, and rapid SCF process speed, producing foods like porridge under various extracting conditions. Each beef sample was extracted with a different pressure (2.03 × 10^7^ Pa, 3.04 × 10^7^ Pa, and 4.05 × 10^7^ Pa), vessel temperature (35 ± 5 °C and 50 ± 5 °C), and different co-solvents (vegetable oil and water) (Figure 2).

**Figure 3:**
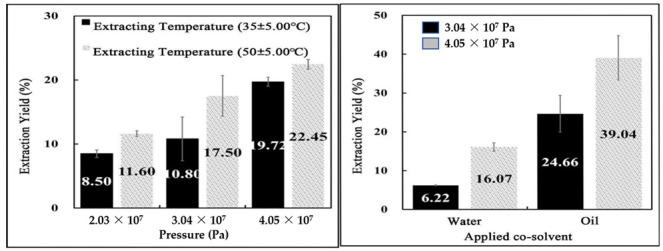
(**Left**)*:* The effect of extracting pressure on beef fat yield rate (%). The fat ingredient from beef samples extracted after supercritical extraction under different pressures (2.03 × 10^7^, 3.04 × 10^7^, and 4.05 × 10^7^ Pa). Two different vessel temperatures were applied as 35 ± 5 °C and 50 ± 5 °C, (**Right**): The effect of co-solvent liquid on beef fat yield rate (%). The fat ingredient from beef samples extracted after supercritical extraction under the same temperature (50 ± 5 °C) but different extracting pressures (3.04 × 10^7^ and 4.05 × 10^7^ Pa) and different co-solvents (vegetable oil and water).

As illustrated in the left of Figure 3, experimental results indicate considerable weight loss in SCF-processed beef. The graph shows the weight reduction after the extraction process, as a function of three different pressures and two temperatures. All SCF-extracted beef showed less mass than the original beef (total mass: 5.0 ± 0.5 g). The presence of fat, protein, cholesterol, moisture, and lipids in the original beef contributes to its mass, and the extraction of fats, cholesterol, and lipids results in a reduced mass percentage (yield). Changes in specific components are not dealt with in detail, as this research focuses on the possibility of printing the processed meat and the conditions for optimization. The extraction yield increases with an increase in temperature and pressure in the SCF extraction process. An SCF extraction temperature of around 35 ± 5 °C indicated increased weight reduction with an increase in pressure (2.03 × 10^7^ Pa: 8.50 ± 0.59%, 3.04 × 10^7^ Pa: 10.80 ± 3.42%, 4.05 × 10^7^ Pa: 19.72 ± 0.70%). The mass of all samples (2.03 × 10^7^ Pa: 11.60 ± 0.46%, 3.04 × 10^7^ Pa: 17.50 ± 3.17%, and 4.05 × 10^7^ Pa: 22.45 ± 0.76%) at the extraction temperature of around 50 ± 5 °C decreased more efficiently than low-temperature extractions. Due to the extraction of the dissolved components in the SCF treatment, the beef samples did not deform significantly in shape, but texture deformation due to drying characteristics was evident.

**Figure 4:**
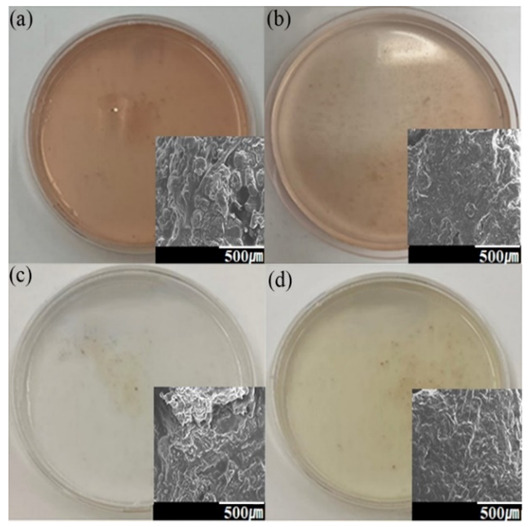
Images of blood diffused in water using SEM for (**a**): unprocessed beef; (**b**): unprocessed halal beef; (**c**): SCF under pressure at 3.04 × 10^7^ Pa processed with water as a co-solvent; and (**d**): SCF under pressure at 3.04 × 10^7^ Pa processed with oil as a co-solvent.

Visually, the beef sample’s color changed to a light brown color after SCF treatment in Figure 2d. The extraction efficiency depends on the type of co-solvents used, and according to the observations, vegetable oil is relatively more effective in extracting lipid than water as a co-solvent (Figure 3, right). SCF with water extracted 6.22 ± 0.10% with an extracting pressure of 3.04 × 10^7^ Pa, and 16.07 ± 1.07% with an extracting pressure of 4.05 × 10^7^ Pa. On the other hand, SCF extraction with vegetable oil decreased mass by 24.66 ± 4.72% at 3.04 × 10^7^ Pa, and 39.04 ± 5.71% at 4.05 × 10^7^ Pa. Thus, SCF treatment with carbon dioxide at high pressure, temperature, and utilizing a vegetable oil co-solvent results in higher lipid and fat loss yield. The experimental results in Figure 4c,d visually confirm lower blood diffusion in water with oil as a co-solvent. Higher pressure improved solvent extraction efficiency by increasing its density and solubility, by utilizing non-polar material as co-solvents.

The Hagen-Poiseuille (HP) equation expressed below includes the following constants. q_w_ = mass flux (g/s), r = the internal radius of the piston or tip (m), ΔP = the pressure drop (Pa), ρ = density of the materials (g/cm^3^), μ = fluid viscosity (Pa·s), and L = the length of the tip or piston (m).

$$q_{w}=\frac{{\pi r}^{4}\Delta P\rho }{8\mu L}$$

A fundamental experiment was conducted to apply the HP to food materials containing water and air with Newtonian behavior and water with relatively high viscosity. Based on an existing experiment, the three components must be sufficiently reviewed to control PTE quantitatively, but real foods are not enough to overcome the configured total pressure drop (∆P_Total_). The applicable water and air results are as follows in Table 3.

**Figure 5:**
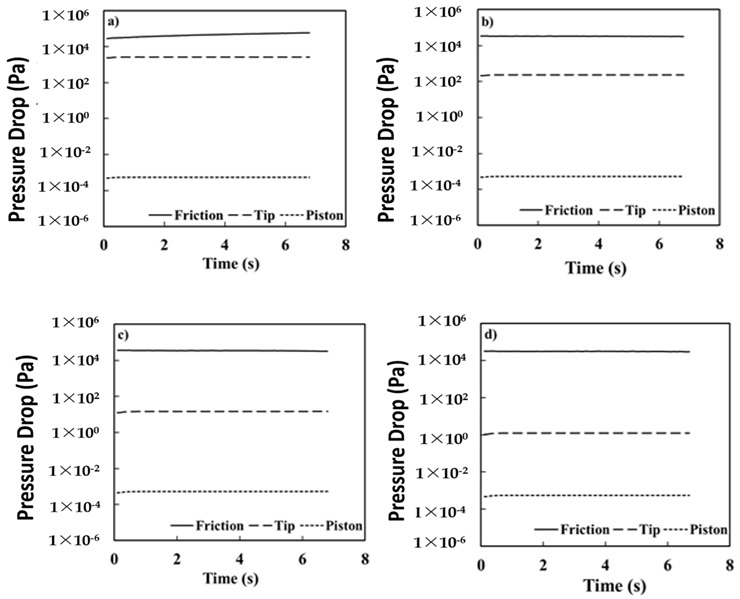
The pressure drop for friction, piston, and tip, for air piston head press at 3.3 × 10^−3^ m/s with various tip sizes; (**a**) 1.1 × 10^−4^ m; (**b**) 2.0 × 10^−4^ m; (**c**) 4.0 × 10^−4^ m; (**d**) 7.5 × 10^−4^ m.

The fundamental experiment involves the printing process, but with just an empty cylinder. The data acquired from the experiment generates primary quantitative spill data for extruding air, which helps understand ΔP_Friction_ as an essential variable. Understanding the pressure drop due to friction is crucial, as the applied force must exceed ΔP_Friction_ for accurate control of the pressure to print different materials. However, ΔP_Friction_ in the experiment is 2.88 × 10^7^ Pa at the smallest tip size available, and is a few hundred times larger than the other two pressure drops (ΔP_Piston_ and ΔP_Tip_). Figure 5 provides empirical data for the part that depended on the HP equation to show the degree of change in the piston’s friction and resistance values, with tip size proving the cylinder is pressurized enough to print with the smallest tip size.

**Figure 6:**
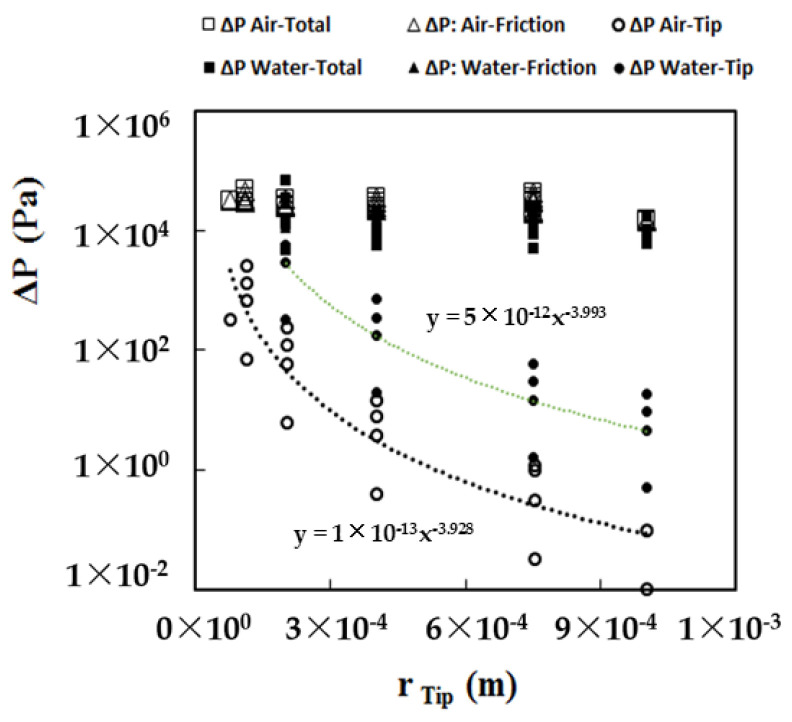
The pressure drop for friction, piston, and tip, for air and water with various tip sizes.

On the other hand, a similar fundamental experiment with water instead of air, with tip sizes 2.0 × 10^−4^, 4.0 × 10^−4^, 7.5 × 10^−4^, and 1.0 × 10^−3^ m, resulted in pressure build-up due to ΔP_Tip_, as water is more viscous than air (Figure 6). Hence, a tip size of 2.0 × 10^−4^ offers precise control for printing as the pressure drop between the tip and the material is significant and cannot be neglected. The observations implied that ΔP_Tip_ is inversely proportional to the 4th power of the radius of the tip (r_Tip_). ΔP_Friction_ increases slightly with the increase in tip size, but ΔP_Tip_ increases rapidly. Hence, the pressure drop between the material and the tip is not negligible. Repetitive experimentations with meat having viscosities several hundred times than that of water proved challenging to discharge quantitatively, irrespective of the meat particles’ size through the largest tip size of 1.0 × 10^−3^ m.

**Figure 7:**
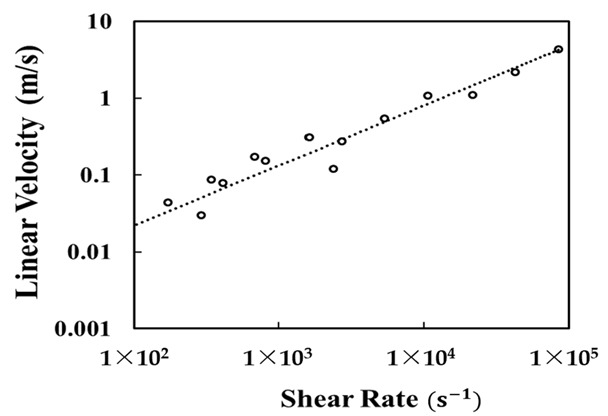
The linear velocity of water vs. various shear rates of PTE.

These experimentations were necessary to understand the quantitative discharge using water, as it is relatable to compare it with foods with high moisture content. The amount of water discharged constantly increases accordingly with the increase in the shear rate applied to the piston. Theoretically, the air and water are separate, but practically, such conditions are unattainable. The air and solid or air and liquid, a high-viscosity substance, behave as a whole. Unlike air, the resistance offered to the material by the tip is significantly affected due to the frictional force between the piston head and the inner wall of the piston. Therefore, the shear rate applied to the piston head increases as the measurement of the high-viscous material flow’s linear velocity is shown in Figure 7. Experimentally, it is expected that meat printing is possible if ingredients such as meat are crushed to a certain level and devoid of lumps. When discharging material containing water and air, the print head speed should be 0.1 m/s and the shear rate should be stable below 1000/s. Since the slurry type meat has a viscosity of several hundred times more, at a shear rate of 1000/s, higher pressure is expected to be applied for stable discharge.

**Figure 8:**
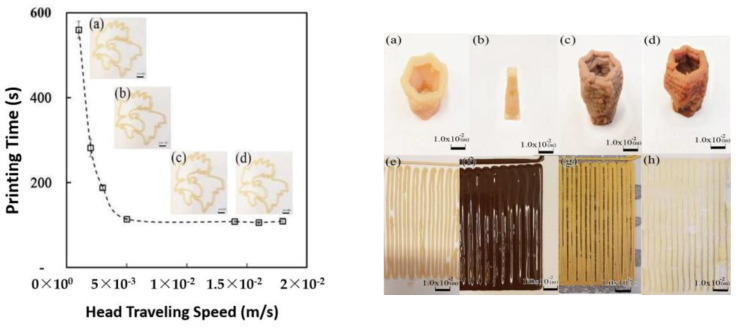
(**Left**)*:* The accuracy of 2.5D printed images as printing time reduces with the increase in head traveling speed. (**a**) Head traveling speed: 1.0 × 10^−3^ m/s; (**b**): head traveling speed: 5.0 × 10^−3^ m/s; (**c**): head traveling speed: 1.5 × 10^−2^ m/s; and (**d**): head traveling speed: 1.8 × 10^−2^ m/s. (**Right**)*:* Images of 2.5D and 3D printed food. (**a**): 3D-printed vase design with Halal chicken (head speed 1.8 × 10^−2^ m/s and tip size 1.0 × 10^−3^ m); (**b**): 3D-printed Chun-Sung-Dae design with Halal chicken (head speed 7.0 × 10^−3^ m/s and tip size 3.2 × 10^−4^ m); (**c**): 3D-printed vase design with SCF beef sample (head speed 1.8 × 10^−2^ m/s and tip size 1.0 × 10^−3^ m); (**d**): 3D-printed vase design with Halal beef (head speed 1. 8 × 10^−2^ m/s and tip size 7.5 × 10^−4^ m); (**e**): 2.5D-printed grid line with SCF-processed beef (head speed 1.8 × 10^−2^ m/s and tip size 1.0 × 10^−3^ m); (**f**): 2.5D-printed grid line with chocolate (head speed 1.8 × 10^−2^ m/s and tip size 1.0 × 10^−3^ m); (**g**): 2.5D-printed grid line with peanut butter (head speed 1.8 × 10^−2^ m/s and tip size 1.0 × 10^−3^ m); and (**h**): 2.5D-printed grid line with potato (head speed 1.8 × 10^−2^ m/s and tip size 1.0 × 10^−3^ m).

The texture of the SCF-treated meat increases the possibility of printing through a 3D printer. The consistency of the SCF meat’s grain size enables the continuous discharge required for stable printing, while minimizing clogging problems. Repeat experiments were performed for two-dimensional high-viscosity printing, obtained by applying a head speed of 1.8 × 10^−2^ m/s and tip size of 1.1 × 10^−3^ m (Figure 8). Optimal conditions for the printing process of the single-line design (SLD) were verified [33]. The gridline design consisting of straight lines has proven to be a useful structure for optimizing 3D-printed food products’ thickness and emissions. When the head speed is in the range of 1.0 × 10^−3^ to 1.8 × 10^−2^ m/s, consistent discharge is evident throughout the printing process. The total time required is ± 1.0 to 1.5 s. A speed of 1.8 × 10^−2^ m/s turned out to be the most stable head speed.

Adopting the Internet of Things (IoT) controlled non-face-to-face and real-time 3D printing systems, we combined multiple distinct patterns simultaneously in the Raspberry Pie controller and Arduino microprocessor with different inks accurately controlled in size and positioning. The inclusion of a web-based 3D printing process enabled data transfer from the University of Texas at El Paso to print food in a facility in Korea University, thereby successfully demonstrating long-distance printing. In Figure 8 (right, a), the 3D printing process using Halal chicken breast with a tip size of 1.0 × 10^−3^ m was implemented, and uniformly discharged 2.14 g for 720 s. As shown in Figure 8 (right, b), the 3D design “Chum-Sung-Dae (ancient astronomical observatory)” was delicately printed, and only discharged 0.42 g for 1200 s. A reduced tip size of 3.2 × 10^−4^ m ensures printing of a more complicated and delicate sized 3D structures. In addition to the significant increase in printability and accuracy for Halal chicken by decreased tip size, head speed reduced from 1.8 × 10^−2^ m/s to 7.0 × 10^−3^ m/s. The printability of foods for the initial 1200 s with a constant force and flow rate was confirmed. The material released at a tip size of 3.2 × 10^−4^ m stacked to a thickness of 4.0 × 10^−4^ m, and the results indicated that layer formation was successful due to the nature of the high-viscosity material of meat sticking to consecutive layers. As shown in Figure 8 (right, c,d), 3D printing with SCF and Halal beef material was also performed. Furthermore, the use of tip size 1.0 × 10^−3^ m verified the printability by discharging 3.03 g for 600 s. The smaller tip (7.5 × 10^−4^ m) was used, and derived the vase’s delicate shape by continuously discharging 2.67 g for 600 s. Moreover, 3D printing experiments with SC-CO_2_ processed beef and other food materials (chocolate, peanut butter, and ground potato) were successful. As seen in the gridline structure of Figure 8 (right, e–h), the 3D printing process was successfully implemented for 160 s.

## 4. Conclusions

The SCF process quickly processes meat to a printable state, further removing fats and cholesterol that could be harmful to the human body, and producing hygienic and customizable ingredients. Printing of halal beef and regular chicken, along with SCF-processed beef, was successful. SCF meat created 3D printable structures with advanced custom materials such as slurry and gel-type texture control. In the SCF process’s meat treatment, the high temperature and extraction pressure increased the process efficiency by 22.5%, and the use of a co-solvent confirmed the effect of reducing meat weight by up to 39.4%. Due to the inherent polarity of the co-solvent, water was effective in extracting residual blood or myoglobin. It has proven to be much more effective in removing fat from meat using co-solvents. These SCFs and the 3D printing process are essential when developing personalized foods that allow fine-tuned nutritional control to meet specific dietary requirements. Printing parameters are optimized for high-viscosity PTE 3D printing of halal meat ingredients and SCF-processed beef. It has identified interest in the food industry and new cooking methodologies, and new possibilities for 3D-printed meat production. In this study, 3D meat printing was successfully attempted in real-time, including long-distance printing over two continents via the web in a non-face-to-face or untact manner.

## Figures and Tables

**Table 1:** Food materials commonly treated using supercritical extraction [22,23].

Materials	Treatment Method	Extracted Ingredients
Vegetables	Temperature range of 35–65 °C and pressure of 4.05 × 107 Pa. Hexane or chloroform as co-solvent.	Extracted carotene from carrots, sweet potatoes, paprika, and tomato paste.
Dairy products	Temperature range of 60–80 °C and pressure of 3.70 × 107~5.47 × 107 Pa. Methanol or ethanol as co-solvents.	Extracted vitamin A and E from cow milk and removing fat.
Eucalyptus leaves	A temperature of 50 °C and pressure of 2.03 × 107 Pa	Extracted plant oil
Coffee beans	A temperature of 90 °C and pressure range of 1.60 × 107~2.20 × 107 Pa. Water as co-solvent.	Decaffeination of green coffee beans
Wine and beer	Temperature range of 25–40 °C and pressure range of 7.60 × 106~3.00 × 107 Pa.	Decreased alcohol concentration

**Table 2:** Supercritical extraction conditions.

SCF Time (min)	Extraction Temperature (°C)	Pressure × 10^7^ (Pa)	Co-Solvent
40	35 ± 5	2.03	-
		3.04	-
		4.05	-
	50 ± 5	2.03	-
		3.04	-
		4.05	-
	50 ± 5	3.04	Water
		4.05	Water
		3.04	Vegetable oil
		4.05	Vegetable oil

**Table 3:** Discharge data for air and water volume and pressure intensification experiment results, according to tip size and press-speed (velocity of the press).

	**Velocity of Press (m/s)**	**Tip Size (m)**	**Shear Rate (1/s)**	**∆P_Friction_** **(Pa)**	**STDEV of ∆P_Friction_**	**∆P_Tip_** **(Pa)**	**STDEV of ∆P_Tip_**	**∆P_Piston_** **(Pa)**	**q_v_** **(m^3^/s)**	**STDEV of q_v_**
Air	8.33 × 10^−5^	4.00 × 10^−4^	2.99 × 10^2^	2.39 × 10^4^	1.83 × 10^3^	4.06 × 10^−1^	2.20 × 10^−1^	1.47 × 10^−5^	1.50 × 10^−8^	8.16 × 10^−9^
	8.33 × 10^−5^	2.00 × 10^−4^	2.34 × 10^3^	2.67 × 10^4^	2.32 × 10^3^	6.39 × 10^0^	2.69 × 10^0^	1.44 × 10^−5^	1.47 × 10^−8^	6.22 × 10^−9^
	8.33 × 10^−5^	1.10 × 10^−4^	1.43 × 10^4^	2.88 × 10^4^	2.60 × 10^3^	7.04 × 10^1^	3.37 × 10^1^	1.45 × 10^−5^	1.49 × 10^−8^	7.13 × 10^−9^
	8.33 × 10^−5^	7.50 × 10^−5^	4.49 × 10^4^	3.04 × 10^4^	1.41 × 10^3^	3.25 × 10^2^	1.49 × 10^2^	1.45 × 10^−5^	1.49 × 10^−8^	6.81 × 10^−9^
	1.67 × 10^−4^	7.50 × 10^−4^	2.94 × 10^2^	4.19 × 10^4^	None	2.13 × 10^−1^	None	9.47 × 10^−5^	9.73 × 10^−8^	None
	3.33 × 10^−4^	7.50 × 10^−4^	5.81 × 10^2^	3.62 × 10^4^	None	4.21 × 10^−1^	None	1.88 × 10^−4^	1.93 × 10^−7^	None
	5.00 × 10^−4^	7.50 × 10^−4^	8.72 × 10^2^	2.98 × 10^4^	None	6.31 × 10^−1^	None	2.81 × 10^−4^	2.89 × 10^−7^	None
	6.67 × 10^−4^	7.50 × 10^−4^	1.14 × 10^3^	1.99 × 10^4^	None	8.26 × 10^−1^	None	3.68 × 10^−4^	3.78 × 10^−7^	None
	8.33 × 10^−4^	1.00 × 10^−3^	1.90 × 10^1^	1.55 × 10^4^	9.20 × 10^2^	1.03 × 10^−2^	1.03 × 10^−2^	1.46 × 10^−5^	1.49 × 10^−7^	7.40 × 10^−9^
	8.33 × 10^−4^	7.50 × 10^−4^	4.52 × 10^1^	2.07 × 10^4^	1.41 × 10^3^	3.27 × 10^−2^	1.63 × 10^−2^	1.46 × 10^−5^	1.50 × 10^−7^	7.44 × 10^−9^
	8.33 × 10^−4^	1.00 × 10^−3^	1.83 × 10^2^	1.44 × 10^4^	1.85 × 10^3^	9.93 × 10^−2^	7.58 × 10^−3^	1.40 × 10^−4^	1.44 × 10^−7^	1.10 × 10^−8^
	8.33 × 10^−4^	7.50 × 10^−4^	4.32 × 10^2^	2.08 × 10^4^	1.30 × 10^3^	3.13 × 10^−1^	2.32 × 10^−2^	1.40 × 10^−4^	1.43 × 10^−7^	1.06 × 10^−8^
	8.33 × 10^−4^	7.50 × 10^−4^	1.41 × 10^2^	1.90 × 10^4^	None	1.02 × 10^0^	None	4.56 × 10^−4^	4.68 × 10^−7^	None
	8.33 × 10^−4^	4.00 × 10^−4^	2.86 × 10^3^	2.13 × 10^4^	1.35 × 10^3^	3.88 × 10^0^	3.17 × 10^−1^	1.40 × 10^−4^	1.44 × 10^−7^	1.17 × 10^−8^
	8.33 × 10^−4^	2.00 × 10^−4^	2.28 × 10^4^	2.50 × 10^4^	1.58 × 10^3^	6.20 × 10^1^	4.91 × 10^0^	1.40 × 10^−4^	1.44 × 10^−7^	1.14 × 10^−8^
	8.33 × 10^−4^	1.10 × 10^−4^	1.38 × 10^5^	2.90 × 10^4^	1.92 × 10^3^	6.80 × 10^2^	6.15 × 10^1^	1.40 × 10^−4^	1.44 × 10^−7^	1.30 × 10^−8^
	1.67 × 10^−4^	7.50 × 10^−4^	8.58 × 10^2^	2.96 × 10^4^	1.28 × 10^3^	1.00 × 10^0^	0.00 × 10^0^	2.77 × 10^−4^	2.84 × 10^−7^	9.92 × 10^−9^
	1.67 × 10^−3^	4.00 × 10^−4^	5.66 × 10^3^	2.94 × 10^4^	8.92 × 10^2^	8.00 × 10^0^	0.00 × 10^0^	2.77 × 10^−4^	2.84 × 10^−7^	1.01 × 10^−8^
	1.67 × 10^−3^	2.00 × 10^−4^	4.51 × 10^4^	2.61 × 10^4^	1.40 × 10^3^	1.23 × 10^2^	4.00 × 10^0^	2.76 × 10^−4^	2.83 × 10^−7^	8.83 × 10^−9^
	1.67 × 10^−3^	1.10 × 10^−4^	2.75 × 10^5^	3.41 × 10^4^	1.16 × 10^3^	1.36 × 10^3^	5.40 × 10^1^	2.80 × 10^−4^	2.87 × 10^−7^	1.14 × 10^−8^
	3.33 × 10^−3^	7.50 × 10^−4^	1.67 × 10^3^	3.14 × 10^4^	5.37 × 10^2^	1.21 × 10^0^	2.76 × 10^−2^	5.40 × 10^−4^	5.55 × 10^−7^	1.26 × 10^−8^
	3.33 × 10^−3^	4.00 × 10^−4^	1.10 × 10^4^	3.47 × 10^4^	9.43 × 10^2^	1.49 × 10^1^	4.43 × 10^−1^	5.38 × 10^−4^	5.53 × 10^−7^	1.64 × 10^−8^
	3.33 × 10^−3^	2.00 × 10^−4^	8.83 × 10^4^	3.31 × 10^4^	5.14 × 10^2^	2.40 × 10^2^	5.68 × 10^0^	5.40 × 10^−4^	5.55 × 10^−7^	1.31 × 10^−8^
	3.33 × 10^−3^	1.10 × 10^−4^	5.33 × 10^5^	4.49 × 10^4^	8.73 × 10^3^	2.63 × 10^3^	4.58 × 10^1^	5.42 × 10^−4^	5.57 × 10^−7^	9.70 × 10^−9^
Water	8.33 × 10^−5^	2.00 × 10^−4^	2.37 × 10^3^	4.83 × 10^3^	3.10 × 10^2^	3.17 × 10^2^	1.06 × 10^1^	6.41 × 10^−4^	1.49 × 10^−8^	4.99 × 10^−10^
	8.33 × 10^−5^	4.00 × 10^−4^	2.95 × 10^2^	5.76 × 10^3^	1.01 × 10^3^	1.91 × 10^1^	3.23 × 10^0^	6.36 × 10^−4^	1.48 × 10^−8^	2.18 × 10^−10^
	8.33 × 10^−5^	7.50 × 10^−4^	4.56 × 10^1^	4.89 × 10^3^	4.15 × 10^2^	1.62 × 10^0^	1.15 × 10^−1^	6.49 × 10^−4^	1.51 × 10^−8^	1.07 × 10^−9^
	8.33 × 10^−5^	1.00 × 10^−3^	1.92 × 10^1^	6.08 × 10^3^	4.06 × 10^2^	5.13 × 10^−1^	5.13 × 10^−1^	6.49 × 10^−4^	1.51 × 10^−8^	1.05 × 10^−9^
	8.33 × 10^−4^	2.00 × 10^−4^	2.17 × 10^4^	1.09 × 10^3^	9.20 × 10^2^	2.89 × 10^3^	1.74 × 10^2^	6.52 × 10^−4^	1.37 × 10^−7^	8.19 × 10^−9^
	8.33 × 10^−4^	4.00 × 10^−4^	2.72 × 10^3^	8.19 × 10^3^	1.64 × 10^3^	1.76 × 10^2^	3.13 × 10^1^	6.54 × 10^−3^	1.36 × 10^−7^	7.63 × 10^−9^
	8.33 × 10^−4^	7.50 × 10^−4^	4.13 × 10^2^	8.73 × 10^3^	8.04 × 10^2^	1.47 × 10^1^	7.41 × 10^−1^	6.55 × 10^−3^	1.37 × 10^−7^	6.90 × 10^−9^
	8.33 × 10^−4^	1.00 × 10^−3^	1.74 × 10^2^	8.45 × 10^3^	9.85 × 10^2^	4.64 × 10^0^	4.64 × 10^0^	6.53 × 10^−3^	1.36 × 10^−7^	7.83 × 10^−9^
	1.67 × 10^−3^	2.00 × 10^−4^	4.29 × 10^4^	2.86 × 10^4^	3.16 × 10^3^	5.71 × 10^3^	5.18 × 10^2^	1.29 × 10^−2^	2.70 × 10^−7^	2.44 × 10^−8^
	1.67 × 10^−3^	4.00 × 10^−4^	5.37 × 10^3^	1.16 × 10^4^	2.43 × 10^3^	3.46 × 10^2^	7.30 × 10^1^	1.29 × 10^−2^	2.70 × 10^−7^	2.14 × 10^−8^
	1.67 × 10^−3^	7.50 × 10^−4^	8.17 × 10^2^	1.10 × 10^4^	8.31 × 10^2^	2.91 × 10^1^	2.18 × 10^0^	1.30 × 10^−2^	2.71 × 10^−7^	2.03 × 10^−8^
	1.67 × 10^−3^	1.00 × 10^−3^	3.45 × 10^2^	1.04 × 10^4^	1.14 × 10^3^	9.22 × 10^0^	9.22 × 10^0^	1.30 × 10^−2^	2.71 × 10^−7^	1.92 × 10^−8^
	3.33 × 10^−3^	2.00 × 10^−4^	8.59 × 10^4^	5.76 × 10^4^	4.54 × 10^3^	1.14 × 10^4^	9.89 × 10^2^	2.57 × 10^−2^	5.40 × 10^−7^	4.65 × 10^−8^
	3.33 × 10^−3^	4.00 × 10^−4^	1.07 × 10^4^	2.10 × 10^4^	2.93 × 10^3^	7.12 × 10^2^	6.38 × 10^1^	2.57 × 10^−2^	5.37 × 10^−7^	4.80 × 10^−8^
	3.33 × 10^−3^	7.50 × 10^−4^	1.63 × 10^3^	1.62 × 10^4^	1.84 × 10^3^	5.79 × 10^1^	4.71 × 10^0^	2.58 × 10^−2^	5.38 × 10^−7^	4.38 × 10^−8^
	3.33 × 10^−3^	1.00 × 10^−3^	6.83 × 10^2^	1.69 × 10^4^	2.21 × 10^3^	1.82 × 10^1^	1.82 × 10^1^	2.57 × 10^−2^	5.36 × 10^−7^	4.64 × 10^−8^

## Data Availability

Data is contained within the article.
